# Prognostic Significance of Abdominal-to-Gluteofemoral Adipose Tissue Distribution in Patients with Breast Cancer

**DOI:** 10.3390/jcm8091358

**Published:** 2019-09-01

**Authors:** Jeong Won Lee, Sung Yong Kim, Hyun Ju Lee, Sun Wook Han, Jong Eun Lee, Sang Mi Lee

**Affiliations:** 1 Department of Nuclear Medicine, International St. Mary’s Hospital, Catholic Kwandong University College of Medicine, 25 Simgok-ro 100 beon-gil, Seo-gu, Incheon 22711, Korea; 2 Department of Surgery, Soonchunhyang University Cheonan Hospital, 31 Suncheonhyang 6-gil, Dongnam-gu, Cheonan, Chungcheongnam-do 31151, Korea; 3 Department of Pathology, Soonchunhyang University Cheonan Hospital, 31 Suncheonhyang 6-gil, Dongnam-gu, Cheonan, Chungcheongnam-do 31151, Korea; 4 Department of Nuclear Medicine, Soonchunhyang University Cheonan Hospital, 31 Suncheonhyang 6-gil, Dongnam-gu, Cheonan, Chungcheongnam-do 31151, Korea

**Keywords:** breast cancer, computed tomography, adipose tissue, prognosis, recurrence

## Abstract

This study aimed to evaluate the association between abdominal-to-gluteofemoral adipose tissue (AT) distribution and recurrence-free survival (RFS) in breast cancer patients. Staging F-18 fluorodexoyglucose (FDG) positron emission tomography/computed tomography (PET/CT) images of 336 women with breast cancer were retrospectively analyzed. From CT images, the volume and CT-attenuation of visceral adipose tissue (VAT), abdominal subcutaneous adipose tissue (SAT), and gluteofemoral AT were measured and the ratio of abdomen-to-gluteofemoral AT volume (AG volume ratio) was calculated. The relationships between adipose tissue parameters and RFS were assessed. Through univariate analysis, abdominal SAT volume, gluteofemoral AT volume, and AG volume ratio were significantly associated with RFS. An increase in abdominal SAT volume and AG volume ratio were associated with an increased risk of recurrence, whereas increased gluteofemoral AT volume was associated with a decreased risk of recurrence. On multivariate analysis, abdominal SAT volume, gluteofemoral AT volume, and AG volume ratio were found to be significant predictors of RFS after adjusting for clinic-histological factors. Irrespective of obesity, patients with a high AG volume ratio showed a higher recurrence rate than those with a low AG volume ratio. Increased abdominal SAT volume and decreased gluteofemoral AT volume were related to poor RFS in breast cancer patients.

## 1. Introduction

Breast cancer is the most commonly diagnosed cancer in women worldwide, accounting for almost 25% of cancer cases according to global cancer statistics [1]. Obesity is a well-known risk factor for the development of breast cancer, and is considered as one of the main causes of rapidly increasing breast cancer incidence rates in developing countries [1,2,3]. Since excessive adipose tissue (AT) in the body can contribute to not only the development but also the progression and metastasis of various malignancies including breast cancer [4,5], the relationship between obesity and breast cancer prognosis has been also widely evaluated. A number of studies have demonstrated the increased risk of recurrence and mortality in obese and overweight patients [6,7,8]. In contrast, some studies have failed to show a significant association between obesity and clinical outcomes in breast cancer patients [9,10,11]. These inconsistent results are thought to result from inaccuracies in the body mass index (BMI) as a measure of body fat amount [12]. Although BMI is the most commonly used scale for defining overweight and obese patients in clinical studies, it cannot distinguish between AT mass and non-fatty tissue mass [12,13]. 

In addition to the amount of AT, several studies have focused on the clinical significance of the distribution of body AT in patients with breast cancer [14,15,16]. Several studies have found that visceral adipose tissue (VAT), abdominal subcutaneous adipose tissue (SAT), and gluteofemoral AT each have distinct genetic, metabolic, and adipokine profiles, which affects their clinical impact on various diseases [13,17,18,19,20]. In diabetes and cardiovascular disease, the amount of VAT and abdominal SAT was positively correlated with disease risk, while gluteofemoral AT played a protective role [17,18]. Since BMI cannot reflect the distribution of AT in the body, the waist-to-hip circumference ratio is used to estimate the central adiposity in clinical studies of malignant diseases [12,14,15]. In previous studies of breast cancer patients, an increased waist-to-hip circumference ratio was found to be associated with poor prognosis, suggesting the prognostic significance of abdominal AT [14,15]. 

Recently, several studies have used imaging techniques such as computed tomography (CT) to assess the amount and distribution of AT, because imaging examinations can provide a more refined method to measure obesity than BMI [12,13,20,21]. Using CT images, the amount of VAT and abdominal SAT can be separately and accurately measured, as well as the total AT [12,13,22]. Furthermore, qualitative features of AT can be assessed through CT-attenuation of AT, expressed as Hounsfield units (HU). The CT-attenuation of AT was found to be positively correlated with inflammatory and fibrotic changes in AT [13,23]. According to previous studies, the volume and CT-attenuation of VAT and abdominal SAT shows a significant association with survival in diverse malignant diseases [13,20,24]. However, in patients with breast cancer, only a few studies have evaluated the prognostic values of measuring the amount and distribution of AT using imaging studies [12,21]. Furthermore, the prognostic significance of gluteofemoral AT measured using CT images has not been assessed. 

In the present study, using non-contrast-enhanced CT images of F-18 fluorodexoyglucose (FDG) positron emission tomography (PET)/CT scans, we measured the volume and CT-attenuation of VAT, abdominal SAT, and gluteofemoral AT, and evaluated whether theses AT parameters showed a significant associations with recurrence-free survival (RFS) in patients with breast cancer. 

## 2. Materials and Methods

### 2.1. Study Population

This study was approved by the Institutional Review Board of Soonchunhyang University, and the requirement to obtain informed consent was waived by the board due to its retrospective nature. All procedures in this study were in accordance with the Declaration of Helsinki. We retrospectively reviewed the electronic medical records of 393 women over 18 years of age who were histopathologically confirmed to have invasive breast cancer and underwent staging FDG PET/CT in Soonchunhyang University Cheonan Hospital between February 2012 and December 2016. Of them, patients who were diagnosed with distant metastasis based on staging examinations (*n* = 18), received a final diagnosis of ductal carcinoma in situ (*n* = 4), had any kind of treatment before the FDG PET/CT scan (*n* = 1), had a previous history of another malignancy or abdominal surgery (*n* = 12), or were lost follow-up within two years after the initial treatment without event (*n* = 7) were excluded from the study. Thus, a total of 351 patients were initially included in the study. During imaging analysis, we excluded a further 15 subjects who had inappropriate CT images for measuring AT parameters due to ascites or metabolic implants in the spine or proximal femur. Therefore, 336 female patients comprised the final study cohort. 

All enrolled subjects underwent pretreatment staging examinations including blood tests, breast ultrasonography, breast magnetic resonance imaging (MRI), FDG PET/CT, and bone scintigraphy. The BMI of each patient was calculated based on the weight and height measured at the time of the staging work-up. Patients were categorized into four groups according to the Asian-Pacific cut-off BMI values: underweight (<18.5 kg/m^2^), normal weight (18.5–23.0 kg/m^2^), overweight (23–25 kg/m^2^), or obesity (>25 kg/m^2^) [25,26]. Clinical TNM stage was determined according to the 7th Edition of the American Joint Committee on Cancer staging system based on the results of imaging examinations. According to the medical records of histopathological evaluation, the status of estrogen receptor (ER), progesterone receptor (PR), human epidermal growth factor receptor 2 (HER2), and Ki67 expression were determined. Tumors were considered positive for ER and PR if they showed 10% or more positively stained cells by immunohistochemistry. Tumors with positive Ki67 expression in 14% or more cells were defined as Ki67-positive tumors. Tumors were considered to be positive for HER2 expression if they showed a 3+ score based on immunohistochemistry or if they showed a 2+ score, but, showing positive gene amplification on fluorescence in situ hybridization. All patients underwent curative surgery for breast cancer with or without neoadjuvant chemotherapy and/or adjuvant treatment according to their tumor stage and clinical condition. After treatments, regular clinical follow-up was performed at intervals of 3–6 months. 

### 2.2. Measurement of Imaging Parameters

FDG PET/CT images of the enrolled patients were obtained with a dedicated PET/CT scanner (Biograph mCT 128 scanner, Siemens Healthcare, Knoxville, TN, USA) from the skull base to the proximal thigh in a supine position after fasting for at least six hours. PET/CT scanning was performed after 60 min uptake period following intravenous injection of approximately 4.07 MBq FDG. Non-contrast-enhanced attenuation correction CT scans were initially performed at 100 mA and 120 kV_p_ imaging with slices at a thickness of 5 mm, followed by PET scans at 1.5 min per bed position. PET images were reconstructed using point-spread-function modeling and time-of-flight reconstruction with attenuation correction.

**Figure 1:**
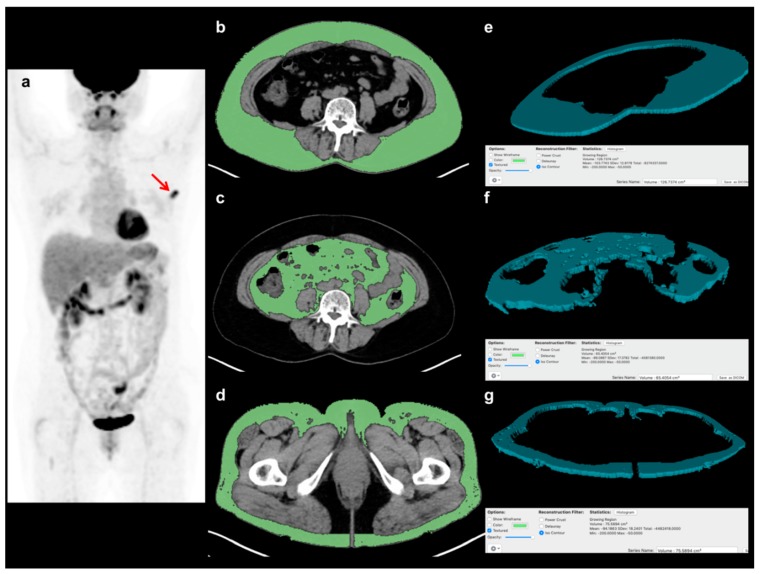
Example of the measurement of volume and CT-attenuation of visceral adipose tissue (VAT), abdominal subcutaneous adipose tissue (SAT), and gluteofemoral adipose tissue (AT). A 57-year-old woman with breast cancer underwent staging FDG PET/CT, showing focally increased FDG uptake at the left breast in a maximum intensity projection image (**a**) (arrow). Using three consecutive transaxial CT images of FDG PET/CT, the areas of VAT, abdominal SAT, and gluteofemoral AT were delineated using a CT-attenuation range of −200 to −50 HU at (**b**) the L4–5 spine level for VAT and (**c**) abdominal SAT, and (**d**) at the proximal femur level for gluteofemoral AT. Based on the areas of each AT in the CT images, (**e**) the three-dimensional structure of VAT, (**f**) abdominal SAT, and (**g**) gluteofemoral AT were automatically created, and their volume and CT-attenuation values were calculated.

A total of six adipose tissue parameters (volume and CT-attenuation of VAT, abdominal SAT, and gluteofemoral AT) were measured from non-contrast-enhanced CT images of FDG PET/CT scans for each enrolled patient without knowledge of the clinical outcomes of the patients. All procedures for adipose tissue measurement were performed with a United States Food and Drug Administration-approved medical image viewer (OsiriX MD 10.0 software, Pixmeo, Geneva, Switzerland). For VAT and abdominal SAT measurements, three consecutive CT image slices at the spinal L4-5 level were selected, because the amount of AT at this level is known to be highly correlated with total body AT volume [27,28]. The abdominal AT area, which is defined as the area with a CT-attenuation range between −200 and −50 HU, was automatically identified in those three slices of CT images. VAT and abdominal SAT were delineated from the abdominal AT area, and the volume and CT-attenuation of VAT and abdominal SAT were measured (Figure 1). For gluteofemoral AT measurement, three consecutive CT image slices at the level of lesser trochanter of the femur were selected. At a CT-attenuation range of −200 and −50 HU, the area of AT was determined in the three CT images. After removing AT from the ischioanal fossa, the volume and CT-attenuation of the AT area were measured and defined as the volume and CT-attenuation of gluteofemoral AT (Figure 1). Based on the volumes of VAT, abdominal SAT, and gluteofemoral AT, the ratio of abdomen-to-gluteofemoral AT volume (AG volume ratio) was calculated as follows: (AG volume ratio) = ((VAT volume) + (abdominal SAT volume)) / (gluteofemoral AT volume). 

Using the fused FDG PET/CT images, a spheroid-shaped volume-of-interest was drawn over the primary breast cancer lesion including the whole cancer lesion. The maximum FDG uptake of the primary cancer lesion, expressed as the standardized uptake value, was measured. 

### 2.3. Statistical Analysis

Spearman rank correlation coefficients were calculated for the volumes of VAT, abdominal SAT, and gluteofemoral AT relative to BMI after evaluating the normality of distribution using the Komogorov-Smirnov test. Differences in volume and CT-attenuation between VAT, abdominal SAT, and gluteofemoral AT were assessed using one-way repeated measures analysis of variance and pairwise multiple comparisons with the Bonferroni correction. The Cox proportional hazard regression test for univariate and multivariate analyses were performed to evaluate the association between RFS and variables including adipose tissue parameters and clinico-histopathologic factors. RFS time was defined as the time from the day of the initial treatment to the day of the detection of cancer recurrence. Patients who did not experience cancer recurrence during follow-up were censored at the day of the last follow-up visit to our medical center. Of the variables used for univariate analysis, those which showed a *p* < 0.1 were included in multivariate survival analysis. In multivariate analysis, the significances of the associations between the adipose tissue parameters and RFS were evaluated after adjusting for age, BMI, and clinco-histopathological factors. For adipose tissue parameters that showed a statistical significance in univariate analysis, receiver-operating-characteristic (ROC) curve analysis was performed to determine the specific optimal cut-off values. Afterwards, survival curves were estimated using the Kaplan-Meier method according to the cut-off values. Cancer recurrence rates between groups were compared using the chi-square test. Statistical analyses were performed using MedCalc Statistical Software version 19.0.3 (MedCalc Software bvba, Ostend, Belgium). A *p* < 0.05 was considered statistically significant.

## 3. Results

### 3.1. Patient Characteristics

The baseline clinical characteristics of the 336 patients enrolled in this study are summarized in Table 1. Among them, 41 patients (12.2%) were histopathologically diagnosed with triple negative breast cancer, and metastatic lymphadenopathy was found in 120 patients (35.7%). Forty-six patients (13.7%) received neoadjuvant chemotherapy, and 332 patients (97.8%) received adjuvant treatment after curative surgery. In all patients, initial treatment was performed within two weeks after FDG PET/CT scans (median, five days). The median duration of clinical follow-up for enrolled patients was 53.3 months (range, 6.1–88.9 months), and, at the time of analysis, recurrence was found in 36 patients (10.7%).

### 3.2. Adipose Tissue Measurement

**Figure 2:**
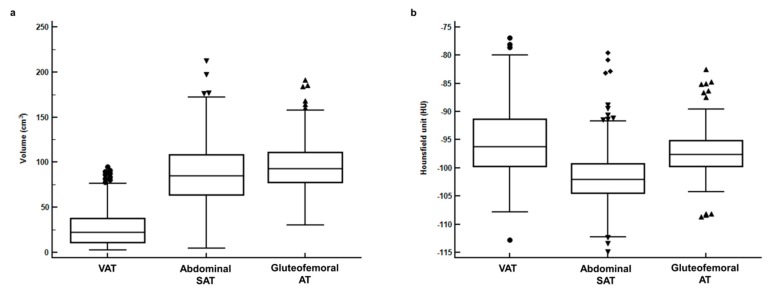
(**a**) Distributions of volume and (**b**) CT-attenuation for visceral adipose tissue (VAT), abdominal subcutaneous adipose tissue (abdominal SAT), and gluteofemoral adipose tissue (gluteofemoral AT).

The BMI showed significant but moderate positive correlations with the VAT (*p* < 0.001, *r* = 0.652, 95% confidence interval (CI) 0.586–0.710), abdominal SAT (*p* < 0.001, *r* = 0.689, 95% CI 0.628–0.741), and gluteofemoral AT (*p* < 0.001, *r* = 0.643, 95% CI 0.576–0.702) volumes. For both volume and CT-attenuation, significant differences were observed between VAT, abdominal SAT, and gluteofemoral AT (*p* < 0.001, Figure 2). In pairwise comparisons, VAT, abdominal SAT, and gluteofemoral AT all showed significant differences in terms of both volume and CT-attenuation (*p* < 0.001 after Bonferroni correction). The gluteofemoral AT volume (mean 96.7 cm^3^, 95% CI 93.2–99.0) showed the highest mean value of all the tissues (mean 27.2 cm^3^, 95% CI 25.1–29.3 for VAT, mean 88.4 cm^3^, 95% CI 85.0–91.8 for abdominal SAT), while the CT-attenuation of VAT (mean −95.4 HU, 95% CI, −96.0–−94.71) showed the highest mean value (mean −101.5 HU, 95% CI −102.0–−101.0 for abdominal SAT, mean −97.3 HU, 95% CI −97.7–−96.9 for gluteofemoral AT). 

### 3.3. Recurrence-Free Survival

The associations between adipose tissue parameters and RFS were assessed using univariate Cox regression analysis, along with the clinico-histopathological factors (Table 2). Among the measured adipose tissue parameters, abdominal SAT volume, gluteofemoral AT volume, and the AG volume ratio were significantly associated with RFS through univariate analysis (*p* < 0.05). An increase in abdominal SAT volume and AG volume ratio was associated with an increased risk of recurrence, with uncorrected hazard ratios of 1.01 (95% CI 1.00–1.02) per 1.0 cm^3^ increase in abdominal SAT volume, and 2.40 (95% CI 1.74–3.29) per 1.0 increase in the AG volume ratio. Conversely, an increase in gluteofemoral AT volume was associated with a decreased risk of recurrence with an uncorrected hazard ratio of 0.97 (95% CI 0.96–0.99) per 1.0 cm^3^ increase in gluteofemoral AT volume. Meanwhile, VAT volume and the CT-attenuation of VAT, abdominal SAT, and gluteofemoral AT showed no significant associations with RFS (*p* > 0.05). Among the investigated clinico-histopathological factors, T stage, N stage, histologic grade, ER status, PR status, Ki67 index, triple negative tumor status, and maximum FDG uptake of the primary tumor were found to be significantly associated with RFS (*p* < 0.05).

Among the investigated AT parameters, abdominal SAT volume, gluteofemoral AT volume, and AG volume ratio, which showed values of *p* < 0.10 through univariate analysis, were selected for multivariate survival analysis (Table 3). In multivariate analysis, the relationship between these adipose tissue parameters and RFS remained significant after adjustment for age, BMI, T stage, N stage, histologic grade, ER status, PR status, Ki67 index, triple negative tumor status, and maximum FDG uptake (*p* = 0.002, hazard ratio 1.02 per 1.0 cm^3^ increase in abdominal SAT volume, *p* < 0.001, hazard ratio 0.98 per 1.0 cm^3^ increase in gluteofemoral AT volume, *p* < 0.001, hazard ratio 2.50 per 1.0 increase in AG volume ratio). 

**Figure 3:**
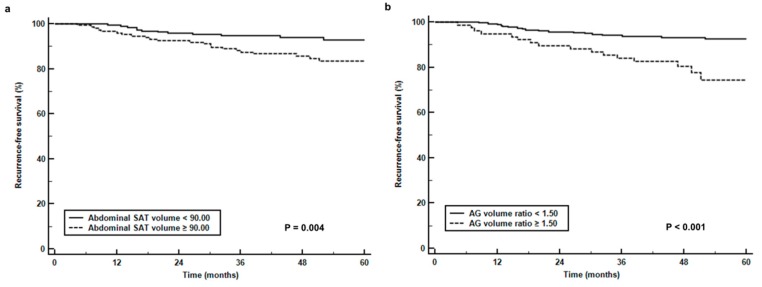

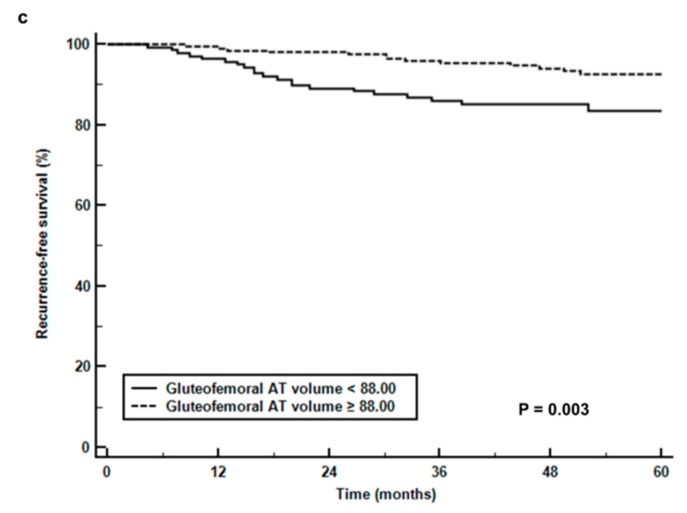
(**a**) Recurrence-free survival curves stratified by abdominal subcutaneous adipose tissue volume (abdominal SAT volume) (**b**), the ratio of abdomen-to-gluteofemoral adipose tissue volume (AG volume ratio), and (**c**) gluteofemoral adipose tissue volume (gluteofemoral AT volume).

For Kaplan-Meier analysis, abdominal SAT volume, gluteofemoral AT volume, and AG volume ratio were dichotomized according to specific cut-off values (90.00 cm^3^ for abdominal SAT volume, 88.00 cm^3^ for gluteofemoral AT volume, and 1.50 for AG volume ratio) as determined by ROC curve analysis. The results of Kaplan-Meier analysis revealed a significant reduction in RFS in patients with a high abdominal SAT volume (*p* = 0.004, 5-year RFS rate, 83.4% vs. 93.0%, Figure 3a) and AG volume ratio (*p* < 0.001, 5-year RFS rate, 74.5% vs. 92.5%, Figure 3b) compared to those with low values and a significant increase in RFS in patients with a high gluteofemoral AT volume (*p* = 0.003, 5-year RFS rate, 92.6% vs. 83.3%, Figure 3c) compared to those with low values. When comparing the recurrence rates based on the combination of BMI and AG volume ratio (Table 4), patients with a high AG volume ratio showed significantly higher recurrence rates than those with a low ratio in both underweight/normal and overweight/obese patients (33.3% vs. 7.9%, *p* = 0.001 for underweight/normal, 18.6% vs. 6.8%, *p* = 0.014 for overweight/obese). 

## 4. Discussion

In the present study, the volumes of abdominal SAT and gluteofemoral AT were found to be significantly associated with RFS in patients with breast cancer. Patients with high abdominal SAT volume showed a decreased RFS, while patients with a high gluteofemoral AT volume showed an increased RFS. The results of our study suggested that the amounts of different specific AT types might have greater prognostic significance rather than the total amount of body AT. 

In recent decades, a number of findings supporting a significant link between AT and cancer cells have found [4,29]. Upon abnormal excess fat accumulation in AT, which is defined as obesity, AT shows dysregulated and altered functions, including the induction of chronic inflammation and insulin resistance, changes in the production of adipokines and cytokines, the promotion of angiogenesis, and extracellular matrix remodeling [4,29]. These disruptions in the activity of AT can contribute to the development, growth, and progression of cancer [4,29,30]. BMI has been used as an indicator to assess body AT for a long time. However, with the increased use of imaging techniques to examine in cancer patients, the area or volume of AT can be more accurately measured by CT images [12,20,21,27]. In various tumor types, the amount of VAT and abdominal SAT were found to have a significant relationship with survival [13,20,31,32,33]. In patients with breast cancer, two studies have evaluated the prognostic value of VAT and abdominal SAT area, as determined by CT imaging [12,34]. One study investigating 172 advanced breast cancer patients showed that patients with a high VAT area had a significantly worse distant disease-free survival [34]. In contrast, another recent study of 3235 breast cancer patients demonstrated a significant positive association between mortality risk and abdominal SAT area, but not VAT area [12]. Similar to their results, we observed that abdominal SAT volume had a significant positive association with RFS, even after adjusting for clinico-histological factors, whereas VAT volume failed to show any significant association. The fact that abdominal SAT shows a stronger correlation with breast AT than VAT or gluteofemoral AT has been suggested as a potential explanation for these results, because breast AT provides an environment that stimulates tumor progression and metastasis, and may display active bi-directional crosstalk with breast cancer cells [12,30,35]. However, because only a few studies have investigated the different effects of abdominal SAT and other AT components, future research should aim to investigate the underlying mechanisms of the different impacts of SAT and other AT types on clinical outcomes [12,20,36]. 

To our knowledge, the present study is the first analysis to evaluate the prognostic significance of gluteofemoral AT volume, as determined by CT imaging in breast cancer patients. In metabolic and cardiovascular diseases, gluteofemoral AT is known to have a major protective role in contrast with VAT and abdominal SAT [18]. Gluteofemoral AT, which is measured based on the hip or thigh circumference or thigh AT mass, is inversely associated with serum cholesterol and insulin levels and is independently associated with a lower risk of diabetes mellitus and coronary heart disease [17,18,19]. Two possible mechanisms have been suggested to explain the protective role of gluteofemoral AT [18]. One is the distinct properties of gluteofemoral AT regarding lipolysis and fatty acid uptake [18,37]. Gluteofemoral AT is thought to protect the body by trapping excessive fatty acids, thereby, preventing chronic lipid exposure [18,37]. Another possible mechanism is that gluteofemoral AT shows differences in its adipokine profiles compared to abdominal AT [18,38]. Although data regarding the differences in adipokine and cytokine secretion between abdominal and gluteofemoral AT remain insufficient, currently, gluteofemoral AT is considered to secrete higher levels of beneficial adipokines and lower levels of inflammatory cytokines than abdominal AT [18,37,38]. In the present study, gluteofemoral AT volume showed significant prognostic value for predicting the recurrence of breast cancer and an improved RFS was observed in patients with a high gluteofemoral AT volume. Given that AT in obese condition contributes to cancer growth by releasing adipokines and inflammatory cytokines and by providing lipids to cancer cells as a source of energy [4,30], gluteofemoral AT might have a protective role against breast cancer. Further studies are needed to validate the results of our study and to elucidate the mechanism of the protective role of gluteofemoral AT. 

Due to increased abdominal SAT volume and decreased gluteofemoral AT volume being associated with a poor RFS, it is no surprise that an increased AG volume ratio was also associated with a poor RFS. The results of our study support the use of waist-to-hip circumference ratio measurements to predict clinical outcomes in patients with breast cancer [14,15]. In our study, patients with a high AG volume had higher recurrence rates than those with a low AG volume irrespective of obesity. This might suggest that the balance between abdominal AT and gluteofemoral AT has a greater significant prognostic impact on breast cancer recurrence than the amount of AT. Patients with central adiposity, or a so-called apple body type, can be considered as having a high risk of recurrence, even if their BMI classification is underweight or normal.

Increased CT-attenuation, measured using non-contrast enhanced CT images, was shown to be associated with smaller adipocytes and increased extracellular matrix fibrosis, which are also found in AT affected by cancer cells [4,23]. In clinical studies of extremity sarcoma, pancreatic cancer, and head and neck cancer, increased CT-attenuation in VAT and abdominal SAT was significantly associated with an increased risk of disease progression and mortality [13,24,28]. Therefore, we evaluated the prognostic value of CT-attenuation in AT in addition to volume. However, the results of our study failed to show any prognostic significance of CT-attenuation in all kinds of AT. Despite this, in the present study, VAT, abdominal SAT, and gluteofemoral AT each showed significant different CT-attenuation values, providing evidence that each of these AT components has distinct features. 

The present study had several limitations that should be mentioned. Firstly, the patient population in our study was retrospectively collected from a single hospital. Therefore, further validation of our results is needed in the general application. Furthermore, the underlying mechanisms linking body AT distribution with poor RFS should be investigated. Additionally, our analysis only assessed body composition in a single CT scan of the patients. A longitudinal follow-up study assessing the changes of body AT distribution with multiple CT scan images might provide important insights regarding the role of AT. Finally, there is still no established method for the proper measurement of the amount and CT-attenuation of AT, which could limit the use of AT parameters in a clinical setting [12,13,21,27,28]. 

## 5. Conclusions

In the present study, the volumes of abdominal SAT and gluteofemoral AT, as measured using pre-treatment CT images were independently associated with RFS in breast cancer. An increase in abdominal SAT volume and AG volume ratio were associated with worse RFS, while an increase in gluteofemoral AT volume was associated with better RFS. The findings in our study demonstrated the prognostic significance of abdominal-to-gluteofemoral AT distribution in breast cancer patients, suggesting a potential distinct relationship between tumor recurrence and abdominal and gluteofemoral AT. Further studies with larger patient populations are warranted to confirm the results of this study. 

## Figures and Tables

**Table 1:** Characteristics of enrolled female patients with breast cancer (*n* = 336).

Characteristics	Number (%)	Median (Range)
Age (years)		51 (30–85)
Body mass index		23.7 (16.4–35.2)
Obesity		
*Underweight/normal*	145 (43.2%)	
*Overweight/obese*	191 (56.8%)	
Menopausal status		
*Premenopausal*	143 (42.6%)	
*Postmenopausal*	193 (57.4%)	
Histopathology		
*Intraductal carcinoma*	299 (89.0%)	
*Intralobular carcinoma*	37 (11.0%)	
T stage		
*T1*	154 (45.8%)	
*T2*	147 (43.7%)	
*T3*	23 (6.8%)	
*T4*	12 (3.6%)	
N stage		
*N0*	216 (64.3%)	
*N1*	69 (20.5%)	
*N2*	29 (8.6%)	
*N3*	22 (6.5%)	
Histologic grade		
*Grade 1*	83 (24.7%)	
*Grade 2*	169 (50.3%)	
*Grade 3*	84 (25.0%)	
Estrogen receptor status		
*Positive*	250 (74.4%)	
*Negative*	86 (25.6%)	
Progesterone receptor status		
*Positive*	208 (61.9%)	
*Negative*	128 (38.1%)	
HER2 status		
*Positive*	169 (50.3%)	
*Negative*	167 (49.7%)	
Ki67 expression status		
*Positive*	221 (65.8%)	
*Negative*	115 (34.2%)	
Maximum FDG uptake		4.05 (1.10–37.90)
VAT volume (cm^3^)		22.1 (5.6–95.0)
VAT CT-attenuation (HU)		−96.3 (−112.8–−76.9)
Abdominal SAT volume (cm^3^)		85.0 (6.8–212.2)
Abdominal SAT CT-attenuation (HU)		−102.0 (−114.9–−79.6)
Gluteofemoral AT volume (cm^3^)		93.3 (30.4–191.1)
Gluteofemoral AT CT-attenuation (HU)		−97.5 (−108.7–−82.5)
AG volume ratio		1.15 (0.40–5.08)
Neoadjuvant chemotherapy		
*Yes*	46 (13.7%)	
*No*	290 (86.3%)	
Adjuvant treatment		
*CTx + RTx + HTx*	162 (48.2%)	
*RTx + HTx*	97 (28.9%)	
*CTx + HTx*	19 (5.7%)	
*CTx + RTx*	5 (1.5%)	
*HTx*	27 (8.0%)	
*CTx*	19 (5.7%)	
*RTx*	3 (0.9%)	
*No*	4 (1.2%)	

HER2, human epidermal growth factor receptor 2; FDG, F-18 fluorodeoxyglucose; VAT, visceral adipose tissue; CT, computed tomography; HU, Hounsfield unit; SAT, subcutaneous adipose tissue; AT, adipose tissue; AG volume ratio: the ratio of abdomen-to-gluteofemoral adipose tissue volume; CTx, chemotherapy; RTx, radiotherapy; HTx, hormonal therapy.

**Table 2:** Univariate analysis for recurrence-free survival.

Variables	*p*-Value	Hazard Ratio (95% CI)
Age (1-year increase)	0.534	1.01 (0.98–1.04)
Obesity (underweight/normal vs. overweight/obese)	0.717	1.13 (0.58–2.18)
Menopausal status (pre vs. post)	0.975	0.99 (0.51–1.92)
T stage		
*T1 stage*	–	1.00
*T2 stage*	<0.001	8.55 (2.56–28.56)
*T3 stage*	<0.001	22.71 (6.32–81.68)
N stage (N0 vs. N1–3)	0.005	2.55 (1.32–4.93)
Histologic grade		
*Grade 1*	–	1.00
*Grade 2*	0.361	0.59 (0.19–1.82)
*Grade 3*	0.001	3.23 (1.59–6.54)
ER status (positive vs. negative)	<0.001	3.76 (1.95–7.25)
PR status (positive vs. negative)	<0.001	4.87 (2.34–10.10)
HER2 status (positive vs. negative)	0.540	0.81 (0.42–1.57)
Ki67 index (negative vs. positive)	0.003	6.07 (1.86–19.78)
Triple negative tumor (no vs. yes)	<0.001	3.54 (1.70–7.36)
Maximum FDG uptake (1.0 increase)	0.001	1.07 (1.03–1.11)
VAT volume (1.0 cm^3^ increase)	0.732	1.00 (0.99–1.02)
VAT CT-attenuation (1.0 HU increase)	0.920	1.00 (0.95–1.06)
Abdominal SAT volume (1.0 cm^3^ increase)	0.006	1.01 (1.00–1.02)
Abdominal SAT CT-attenuation (1.0 HU increase)	0.273	1.04 (0.97–1.12)
Gluteofemoral AT volume (1.0 cm^3^ increase)	<0.001	0.97 (0.96–0.99)
Gluteofemoral AT CT-attenuation (1.0 HU increase)	0.135	1.07 (0.98–1.17)
AG volume ratio (1.0 increase)	<0.001	2.40 (1.74–3.29)

CI, confidence interval; ER, estrogen receptor; PR, progesterone receptor;HER2, human epidermal growth factor receptor 2; FDG, F-18 fluorodeoxyglucose; VAT, visceral adipose tissue; CT, computed tomography; HU, Hounsfield unit; SAT, subcutaneous adipose tissue; AT, adipose tissue; AG volume ratio: the ratio of abdomen-to-gluteofemoral adipose tissue volume

**Table 3:** Multivariate models for recurrence-free survival.

Variables	Model with Abdominal SAT Volume		Model with Gluteofemoral AT Volume		Model with AG Volume Ratio	
	*p*-Value	Hazard Ratio (95% CI)	*p*-Value	Hazard Ratio (95% CI)	*p*-Value	Hazard Ratio (95% CI)
T stage						
*T2*	0.003	6.46 (1.91–21.79)	0.002	6.82 (2.02–23.07)	0.005	5.92 (1.74–20.20)
*T3* *–* *4*	<0.001	18.26 (4.95–67.36)	<0.001	15.39 (4.11–57.66)	<0.001	16.04 (4.22–61.05)
N stage	0.913	1.04 (0.50–2.19)	0.720	1.15 (0.54–2.42)	0.834	0.92 (0.44–1.95)
Histologic grade						
*Grade 2*	0.243	2.01 (0.62–6.50)	0.516	1.46 (0.54–2.42)	0.076	3.41 (0.88–13.25)
*Grade 3*	0.120	2.59 (0.79–8.45)	0.180	1.68 (0.79–3.59)	0.027	4.95 (1.20–20.47)
Estrogen receptor	<0.001	3.65 (1.75–7.63)	0.010	2.92 (1.29–6.63)	0.043	2.41 (1.03–5.66)
Progesterone receptor	0.094	2.53 (0.85–7.51)	0.048	2.85 (1.01–8.07)	0.293	1.87 (0.58–6.03)
Ki67 index	0.306	1.95 (0.54–7.03)	0.270	2.07 (0.57–7.50)	0.435	1.67 (0.46–6.09)
Triple negative tumor status	0.547	1.34 (0.52–3.47)	0.822	0.894 (0.34–2.38)	0.756	1.16 (0.46–2.93)
Maximum FDG uptake	0.600	0.98 (0.93–1.05)	0.483	0.98 (0.92–1.04)	0.586	0.98 (0.92–1.05)
Abdominal SAT volume	0.002	1.02 (1.01–1.03)				
Gluteofemoral AT volume			<0.001	0.98 (0.96–0.99)		
AG volume ratio					<0.001	2.50 (1.64–3.81)

All models were adjusted for age and body mass index. FDG, F-18 fluorodeoxyglucose; SAT, subcutaneous adipose tissue; AT, adipose tissue; AG volume ratio: the ratio of abdomen-to-gluteofemoral adipose tissue volume; CI, confidence interval.

**Table 4:** Recurrence rates based on the combination of BMI and AG volume ratio.

		BMI	
		Underweight/Normal (BMI < 23.0 kg/m^2^)	Overweight/Obesity (BMI > 23.0 kg/m^2^)
AG volume ratio	<1.50	10/127 (7.9%)	9/132 (6.8%)
	>1.50	6/18 (33.3%)	11/59 (18.6%)
	*p*-value	0.001	0.014

BMI, body mass index; AG volume ratio: the ratio of abdomen-to-gluteofemoral adipose tissue volume.
